# AI teaching innovation behavior among college teachers: a structural equation modeling analysis based on the AI-TPACK framework, teaching self-efficacy, professional identity, and AI literacy

**DOI:** 10.3389/fpsyg.2026.1834827

**Published:** 2026-09-07

**Authors:** Xue Bai, Tsung-Shun Hsieh

**Affiliations:** 1 Department of Education, Xinzhou Normal University, Xinzhou, Shanxi, China; 2 Asia Innovation Institute, Assumption University of Thailand, Bangkok, Thailand

**Keywords:** AI literacy, AI teaching innovation behavior, AI-TPACK, multi-group analysis, professional identity, teaching self-efficacy

## Abstract

**Background:**

As generative artificial intelligence (GenAI) increasingly enters higher education, understanding how teachers integrate GenAI into innovative teaching is critical. Prior research has mainly focused on intentions to use AI tools. Guided by the AI-Technological Pedagogical Content Knowledge (AI-TPACK) framework, Social Cognitive Theory, and professional identity theory, this study explored the relationships surrounding GenAI-supported teaching innovation behavior, with a focus on teaching self-efficacy, professional identity, and AI literacy.

**Methods:**

A total of 898 Chinese university teachers from eight comprehensive universities completed standardized scales assessing AI-TPACK dimensions, teaching self-efficacy, professional identity, AI literacy, and AI teaching innovation behavior. Data were analyzed via structural equation modeling and multi-group analysis.

**Results:**

The results showed that among the AI-TPACK dimensions, all except AI technological knowledge and integrative knowledge were significantly associated with AI teaching innovation behavior. Professional identity and AI literacy partially mediated the relationship between competence structures and innovative behavior, whereas the mediating effect of teaching self-efficacy was only partially supported. Further multi-group analysis showed that the relationship between professional identity and innovative behavior was stronger among teachers with lower reported frequencies of GenAI tool use.

**Discussion:**

These findings provide empirical evidence on the associations of competence- and psychology-related factors with GenAI-supported teaching innovation. The study highlights the roles of professional identity, AI literacy, and teaching self-efficacy in promoting innovative teaching and offers practical implications for faculty development and AI integration in higher education.

## 1 Introduction

As GenAI becomes widely applied in higher education, teaching is shifting from a stage of “technology assistance” to one of “intelligent reconstruction” (Ma and Chen, 2024). Unlike earlier educational technologies that primarily functioned as tools for information delivery, management, or communication, GenAI can generate instructional content, provide adaptive feedback, and participate in teaching processes through human–AI collaboration. These capabilities are reshaping how teachers design, implement, and evaluate teaching activities (Zheng and Chen, 2025). In this shift, college teachers are no longer simply users of technology. They increasingly serve as designers and practitioners of GenAI-supported instructional innovation. Compared with earlier studies that emphasized teachers’ technology use intention or adoption intention, recent research has begun to examine how teachers actively integrate GenAI into authentic teaching contexts to implement innovative instructional practices (Gao and Yang, 2026; Liu-Schuppener, 2023; Wang Q. et al., 2025; Wang et al., 2025b; Zhao et al., 2026). As GenAI systems become increasingly capable of generating content and supporting instructional decision-making, teachers are required not only to use technology but also to collaborate with AI in teaching processes. Understanding this process has become a central issue in research on educational digitalization (Lu et al., 2024; Wu et al., 2024).

Previous studies have primarily relied on the Technology Acceptance Model (TAM) and the Unified Theory of Acceptance and Use of Technology (UTAUT) to explain teachers’ intentions to use emerging technologies (Lu et al., 2024; Zhang and Wareewanich, 2024). These models mainly examine the link between cognitive evaluations and behavioral intentions. However, they pay limited attention to how teachers transform technology into innovative teaching practices specifically in GenAI-supported contexts. In the context of GenAI, teachers must do more than understand the technology itself. They need the capacity to embed AI into subject knowledge and instructional strategies in meaningful ways. Traditional technology acceptance frameworks therefore do not fully capture AI teaching innovation behavior (AI-TIB). AI-TIB refers to teachers’ self-reported efforts to generate and implement innovative practices in GenAI-supported instruction, including evaluating AI-generated outputs, adapting them to pedagogical goals, and co-designing learning activities with AI systems.

Against this background, the AI-Technological Pedagogical Content Knowledge (AI-TPACK) framework provides a context-specific lens for understanding teachers’ AI-related competence structures, including their knowledge of AI technologies, pedagogical applications, and disciplinary integration in AI-supported environments (Ning et al., 2024). The emergence of GenAI has fundamentally altered the knowledge requirements associated with instructional innovation. In traditional technology-enhanced teaching, technology typically served as a supplementary instructional resource. In contrast, GenAI actively participates in content generation, instructional design, assessment support, and teaching decision-making. Consequently, teachers must not only know how to use AI tools but also evaluate AI-generated content, understand the implications of algorithm-driven outputs, and effectively coordinate human–AI collaboration in pedagogically appropriate ways (Bin-Nashwan et al., 2025). Extending the traditional TPACK model, AI-TPACK highlights teachers’ understanding of AI characteristics, algorithmic logic, ethical issues, and human–AI collaboration. It also emphasizes their ability to integrate technology, pedagogy, and subject content in AI contexts. Compared with TPACK, AI-TPACK offers a more suitable framework for understanding how teachers turn GenAI into pedagogically meaningful innovation rather than merely using it as a tool. It also helps clarify the knowledge base of college teachers’ AI-TIB in GenAI-supported settings.

However, knowledge alone does not guarantee innovation. Social Cognitive Theory (SCT) suggests that behavior depends not only on competence but also on self-efficacy beliefs and cognitive appraisal (Bandura, 1989). Accordingly, possessing AI-related knowledge may not directly translate into innovative teaching practices unless teachers develop sufficient confidence, cognitive understanding, and motivation to apply such knowledge in practice. In AI-supported teaching, technological complexity may weaken teachers’ sense of control and reduce their willingness to experiment. Teaching self-efficacy (TSE) may therefore represent a psychological pathway through which AI-TPACK is translated into innovative practice, as teachers who feel more capable of managing AI-related instructional challenges are more likely to experiment with new teaching approaches (Abbitt, 2011). Similarly, AI literacy (AIL) reflects teachers’ ability to critically understand, appropriately apply, and ethically evaluate AI technologies, enabling them to transform AI-related knowledge into meaningful instructional applications (Ng et al., 2021). Professional identity theory (PIT) further suggests that teachers’ identification with their professional roles shapes their responses to educational change (Beijaard et al., 2004). From this perspective, professional identity (PI) provides a motivational mechanism linking competence to innovation, because teachers who perceive AI integration as consistent with their professional values are more likely to actively engage in AI-related instructional transformation (Su, 2023). Teachers with stronger PI are more likely to view AI integration as professional growth rather than a threat, and thus engage more actively in innovative practice.

In summary, prior research shows three main limitations. First, most studies focus on technology adoption, AI use, or technology integration, while relatively little attention has been paid to AI-TIB. Unlike AI use, adoption, or integration, AI-TIB emphasizes teachers’ self-reported generation and implementation of innovative teaching practices in GenAI-supported instructional contexts. Second, although TPACK has been widely used to explain technology integration in education, it does not fully capture the distinctive knowledge requirements associated with GenAI, such as evaluating AI-generated content, understanding algorithmic processes, and managing human–AI collaborative teaching practices. Third, although existing studies have linked technology-related competencies to educational technology use, it remains empirically unclear how AI-TPACK dimensions are associated with AI-TIB and whether this relationship operates through teachers’ AI literacy, teaching self-efficacy, and professional identity. Scholars have not systematically explained how knowledge structures are associated with innovative behavior in relation to cognitive beliefs and professional identity.

To address these gaps, this study adopts AI-TPACK as the core theoretical framework and integrates SCT and PIT. We propose a multi-level mediation model in which college teachers’ AI-TPACK relates to AI-TIB in GenAI-supported teaching contexts through AIL, TSE, and PI. Through this integrative framework, we aim to clarify the dynamic process linking knowledge structure, cognitive beliefs, PI, and innovative behavior. The findings provide theoretical support and practical implications for teacher professional development in the context of digital transformation in higher education.

## 2 Theoretical framework and research hypotheses

### 2.1 Theoretical framework

**Figure 1:**
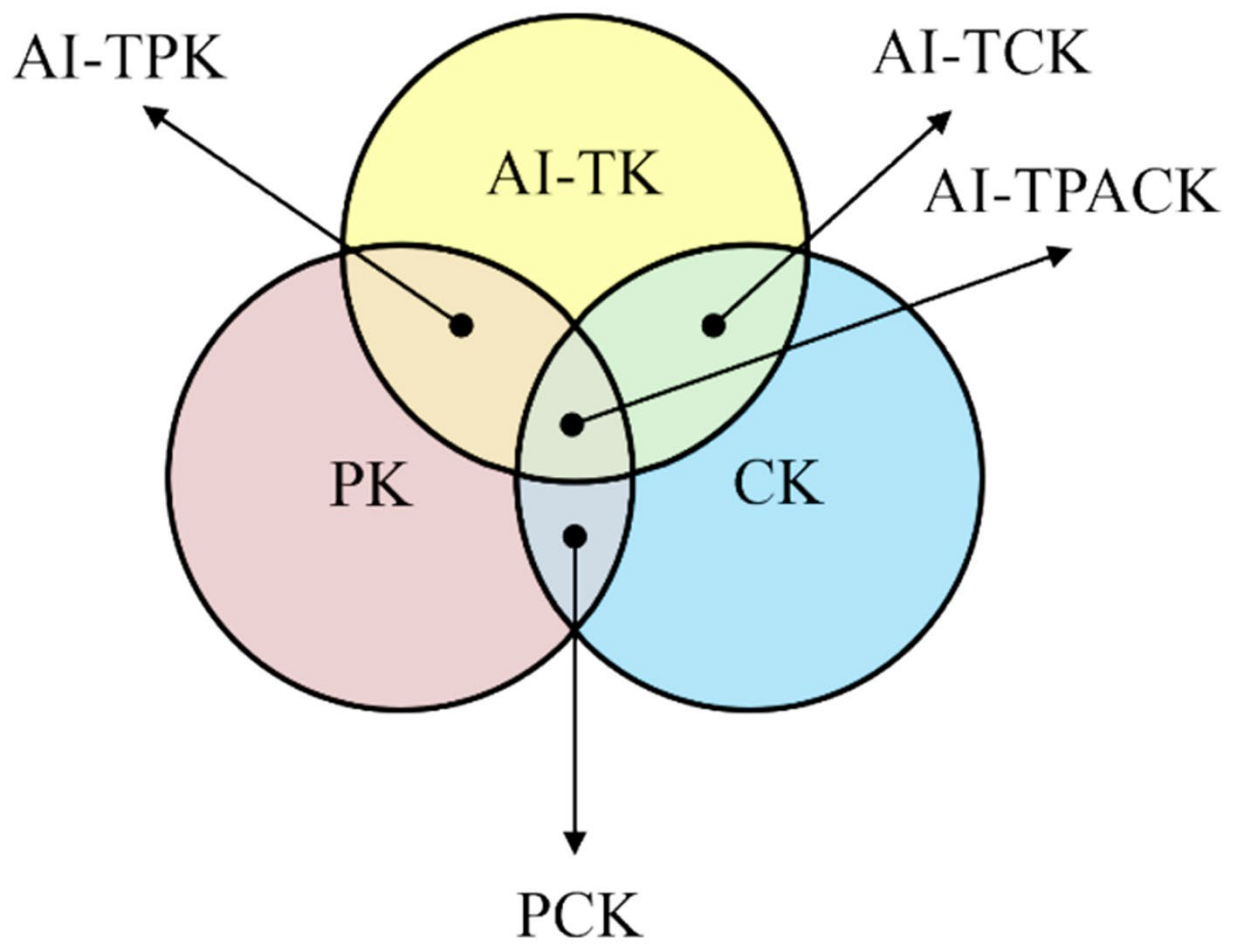
AI-TPACK framework structural diagram.

As AI becomes deeply embedded in higher education, the traditional TPACK framework no longer fully explains teachers’ knowledge structures in GenAI contexts. Unlike conventional educational technologies, GenAI actively generates instructional content, provides adaptive feedback, and participates in instructional decision-making through human–AI collaboration, fundamentally altering teachers’ knowledge and behavioral requirements for innovation (Lee and Moore, 2024; Li, 2025). To address this gap, this study adopts AI-TPACK framework as the core analytical framework. AI-TPACK extends TPACK by emphasizing teachers’ AI-related competence structures, including their understanding of AI mechanisms, data characteristics, algorithmic processes, ethical boundaries, and their ability to integrate AI with pedagogy and subject content. Within this framework, AI-related competence is conceptualized as seven interconnected knowledge domains. Content Knowledge (CK) and Pedagogical Knowledge (PK) reflect teachers’ disciplinary and pedagogical foundations. AI-Technological Knowledge (AI-TK) refers to knowledge of AI principles and applications. Pedagogical Content Knowledge (PCK) captures the integration of pedagogy and subject content. AI-Technological Content Knowledge (AI-TCK) emphasizes the use of AI within disciplinary contexts. AI-Technological Pedagogical Knowledge (AI-TPK) focuses on aligning AI tools with instructional strategies. And AI-Technological Pedagogical Content Knowledge (AI-TPACK) represents teachers’ integrative capacity to coordinate technology, pedagogy, and content in complex teaching situations (Ning et al., 2024). Figure 1 illustrates the structure of the AI-TPACK framework and the relationships among its competence dimensions. Unlike the traditional TPACK framework, which often explains technology adoption or usage intention, AI-TPACK better captures how teachers implement innovative practices in authentic teaching contexts.

Although the AI-TPACK framework could theoretically be modeled as a higher-order construct, this study retains the seven dimensions separately because different knowledge domains may contribute differently to teachers’ AI-related competencies and innovative practices. Examining these dimensions independently enables a more precise understanding of how specific competence domains relate to AI teaching innovation behavior and its psychological mechanisms.

However, knowledge structures do not automatically translate into behavioral outcomes. According to SCT, behavior depends not only on competence but also on self-efficacy beliefs and cognitive appraisal processes. TSE reflects teachers’ confidence in managing instructional tasks (Tschannen-Moran and Hoy, 2001), while AIL denotes their capacity to critically understand, properly use, and ethically assess AI tools. In AI-rich environments characterized by complexity and uncertainty, these cognitive factors may serve as key mediators linking AI-TPACK to AI-TIB. In other words, AI-TPACK is more likely to relate to innovative practice when teachers possess sufficient AIL and strong TSE (Al-Abdullatif, 2024; Joo et al., 2018).

PIT also provides a useful perspective for understanding teachers’ behavioral choices during technological change. PI refers to the extent to which individuals identify with their professional roles, core values, and sense of mission. As AI reshapes the boundaries of the teaching profession, PI may be related to teachers’ engagement in AI-related innovation. Teachers with stronger PI are more likely to view AI integration as an opportunity for professional growth rather than a threat, and thus show greater willingness to implement innovative practices.

Based on this theoretical integration, we propose a multi-level mediation model. The model assumes that AI-TPACK serves as a cognitive foundation and relates to AI-TIB through AIL, TSE, and PI. This pathway links knowledge structures, cognitive beliefs, professional identity, and human–AI collaborative practices, explaining how teachers’ AI-TPACK translates into innovative behavior in GenAI contexts.

### 2.2 AI-TPACK and AI teaching innovation behavior

Innovative behavior refers to the extent to which organizational members generate and implement new ideas to enhance performance (Lin and Shin, 2021). In education, teaching innovation behavior reflects teachers’ proactive efforts to introduce and apply novel and effective instructional ideas, methods, or technologies (Vermeulen et al., 2022; Wu et al., 2022). Prior research has examined the associations between TPACK and both teaching innovation intention and behavior. As an integrative representation of technological, pedagogical, and content knowledge, TPACK has been linked to teachers’ capacity to implement structural instructional reform (Tang et al., 2023). For example, An et al. (2022) drawing on UTAUT and TPACK, found that TPACK dimensions positively predicted Chinese secondary English teachers’ intentions to use AI in teaching. Li et al. (2024) reported that mathematics teachers with higher TPACK were more likely to integrate learning models with technology-based media and to show greater initiative in curriculum redesign and instructional planning. From a theoretical perspective, innovation relies on cross-domain integration and contextual restructuring. It involves redesigning instructional logic rather than simply replacing tools. TPACK highlights the dynamic integration of technology, pedagogy, and content, which enhances teachers’ holistic understanding of teaching contexts and their ability to redefine instructional problems (Bai et al., 2024; Su, 2023). Such integrative knowledge reduces cognitive fragmentation between technology and pedagogy and supports flexible solution generation.

In the era of Generative AI, technological environments have changed substantially, but the logic of integration remains central. AI-TPACK extends TPACK by specifying AI-related knowledge and emphasizing the deep embedding of AI within disciplinary content and instructional design (Casal-Otero et al., 2023). Unlike conventional digital technologies that primarily support information delivery or task completion, GenAI can generate instructional content, provide adaptive responses, and participate in teaching processes through human–AI collaboration (Bhatt et al., 2026; Lee and Moore, 2024; Wang et al., 2025a; Wang Z. et al., 2026). Consequently, instructional innovation in GenAI-supported contexts increasingly depends on teachers’ ability to critically evaluate AI-generated outputs, align algorithm-driven recommendations with pedagogical goals, and effectively coordinate human–AI interactions within classroom settings. These capabilities highlight how the generative, algorithmic, and collaborative nature of GenAI reshapes teachers’ knowledge requirements and is related to their innovative practices, reinforcing the value of an integrative AI-TPACK framework.

Based on prior theoretical and empirical evidence linking TPACK to teaching innovation behavior, and considering the enhanced demands for technology integration in AI-supported contexts, it is reasonable to expect that AI-TPACK, as an integrative knowledge structure, will be positively associated with college teachers’ AI-TIB. Accordingly, we propose the following hypothesis:

*H1*: Each dimension of AI-TPACK is positively associated with college teachers’ AI-TIB.

### 2.3 Relationships among professional identity, AI literacy, and teaching self-efficacy

PI refers to individuals’ recognition, acceptance, and emotional commitment to their occupation, reflecting their self-concept, value affiliation, and willingness for sustained engagement in their professional role (Beijaard et al., 2004). In the context of teacher education, PI specifically refers to the extent to which teachers identify with their professional roles, educational mission, and professional community (Beijaard et al., 2004; Karaolis and Philippou, 2019). Several studies indicate that PI is positively associated with TSE (Canrinus et al., 2012; Moslemi and Habibi, 2019; Yao et al., 2021). According to Bandura (1989)’ SCT, when teachers strongly identify with their professional role, they form a positive self-concept, which enhances their confidence in addressing instructional challenges. PI satisfies teachers’ needs for belonging and achievement, thereby motivating their instructional engagement. For instance, Cai et al. (2022) found that teachers’ PI is positively associated with their self-efficacy, which in turn is related to teaching practices. In GenAI-supported contexts, teachers with higher PI are particularly likely to engage confidently with AI-mediated tasks and handle AI-generated outputs effectively. This study posits that teachers with higher PI may show higher TSE.

Long and Magerko (2020) defined AIL as “a set of abilities that enable individuals to critically evaluate AI technologies, communicate and collaborate effectively with AI, and use AI as a tool across online, home, and workplace environments.” In GenAI-supported settings, AIL includes the ability to evaluate AI-generated content, interpret algorithmic recommendations, and coordinate human-AI collaboration (Allen and Kendeou, 2024). Although direct empirical evidence linking AIL to TSE remains limited, previous research on AI-related competence and technology literacy suggests that teachers’ knowledge and skills in emerging technologies contribute to their confidence and capability in technology-supported teaching (Cosby et al., 2023; Jeon and Kim, 2022). From the perspective of SCT, such competence may enhance teachers’ perceived control and mastery experiences when engaging with AI-supported instructional environments, thereby strengthening TSE (Bandura, 1989). Teachers with higher AIL may better understand AI functions, evaluate AI-generated outputs, and integrate AI resources into instructional design, thereby reducing uncertainty and enhancing their confidence in AI-supported teaching. Moreover, previous studies on technology-related competence have shown that digital literacy and technological capabilities are associated with teachers’ professional development and PI (Burnett, 2011; Su, 2023; Wang and Ye, 2025). In GenAI contexts, AIL may extend this mechanism by helping teachers perceive themselves as competent participants in AI-mediated instructional innovation. For example, Wang and Ye (2025) found that teachers’ digital literacy was associated with PI, suggesting that technology-related competence may contribute to teachers’ professional self-perceptions during educational transformation. Derived from these discoveries, we formulate these hypotheses:

*H2*: PI is positively associated with college teachers’ TSE.

*H3*: AIL is positively associated with college teachers’ TSE.

*H4*: AIL is positively associated with college teachers’ PI.

### 2.4 The mediating role of teaching self-efficacy

Self-efficacy refers to individuals’ beliefs in their ability to organize and execute actions required to achieve specific performance levels (Bandura, 1982). In educational contexts, TSE reflects teachers’ confidence in effectively completing instructional tasks and influencing student learning outcomes. According to SCT, efficacy beliefs develop from individuals’ integration of professional competence and their appraisal of task complexity (Bandura, 1982). In GenAI-supported teaching environments, teachers are expected not only to operate AI tools but also to understand their generative processes, algorithmic logic, and potential risks, and to integrate these capabilities with subject content and instructional strategies. AI-TPACK thus represents an important competence foundation for teaching in AI-supported contexts. Empirical studies have reported positive associations between AI-TPACK and teachers’ self-efficacy (Erol et al., 2025; Xu et al., 2025). Higher AI-TPACK relates to stronger perceived control and competence in AI-based teaching practice (Du et al., 2025). From a theoretical standpoint, AI-TPACK may strengthen teachers’ ability to interpret AI-supported teaching situations, manage GenAI-related uncertainty, and reduce cognitive burden. When teachers understand GenAI logic and integrate it effectively into instruction, they are more likely to experience successful practice and positive feedback. Such mastery experiences form an important source of TSE (Abbitt, 2011). Thus, AI-TPACK functions not only as a knowledge base but also as a cognitive and practical resource that strengthens teachers’ confidence in applying GenAI in instruction.

Prior research shows that TSE is closely related to teachers’ innovative classroom practices (Davies, 2013; Kao et al., 2020). TSE reflects teachers’ confidence in organizing instruction, managing classroom challenges, and promoting student learning. This belief shapes not only attitudes but also behavioral choices and persistence. Teachers with stronger TSE are more likely to adopt new strategies, experiment with new methods, and remain proactive during instructional reform (Huang et al., 2025). For example, Long and Waugh (2018) found that self-efficacy significantly predicted teachers’ decisions to adopt flipped classrooms. From the perspective of SCT, self-efficacy influences how individuals appraise task difficulty, invest effort, and persist when facing obstacles (Bandura, 1982). In GenAI-supported teaching environments, the generative and algorithmic features of GenAI may increase uncertainty in instructional control, making efficacy beliefs especially important. Teachers with higher TSE are therefore more likely to maintain confidence and integrate AI effectively into course design and classroom interaction, thereby engaging in AI-TIB. Recent studies have also emphasized the mediating role of self-efficacy in translating resources into professional practice (Bai et al., 2024; Huang et al., 2020; Huang et al., 2025). For instance, Cai and Tang (2021) found that teachers’ self-efficacy partially mediated the association between school resources and teaching innovation. Bai et al. (2024) reported that self-efficacy mediated the link between TPACK and technology use intention. These findings suggest that professional competence alone does not directly lead to innovation, and that efficacy beliefs may help explain this association. In GenAI contexts, AI-TPACK can be viewed as a professional competence resource. Whether teachers translate this resource into AI-TIB may depend on their confidence in applying it. TSE may therefore serve as a key explanatory factor in the association between AI-TPACK and AI-TIB. Accordingly, we propose the following hypotheses:

*H5*: Each dimension of AI-TPACK is positively associated with college teachers’ TSE.

*H6*: TSE is positively associated with college teachers’ AI-TIB.

*H7*: TSE mediates the associations between AI-TPACK dimensions and AI-TIB.

### 2.5 The mediating role of professional identity

Prior research indicates that teachers’ technology integration knowledge relates closely to the development of PI. Empirical evidence shows that higher levels of TPACK associate with stronger PI (Golzar et al., 2023; Mohammadkarimi, 2023; Su, 2023). For example, Golzar et al. (2023) found positive associations between all TPACK dimensions and teachers’ PI. As GenAI rapidly reshapes teaching tasks, instructional decision-making, and professional boundaries, teachers’ identities undergo adaptation and reconstruction (Lan, 2024). In this context, AI-TPACK highlights teachers’ ability to understand AI technologies, critically evaluate AI-generated outputs, and integrate AI with pedagogy and subject content. Building on prior findings linking technology integration competence to PI, it is reasonable to expect that higher AI-TPACK relates to stronger professional control and competence perceptions in GenAI-supported teaching. This experience may help teachers interpret technological change as an opportunity for growth rather than a threat, thereby strengthening PI and supporting their transition toward roles such as instructional designers and learning facilitators in the AI era.

Although few studies have directly examined the link between PI and TIB among teachers, research in broader organizational contexts has consistently shown that PI relates to individual innovative behavior (Lin and Chang, 2022; Zhu et al., 2025). Individuals with stronger PI tend to demonstrate greater role commitment and professional engagement, which increases their willingness to propose and implement new ideas in practice (Lin and Chang, 2022). From the perspective of PIT, when individuals incorporate a professional role into their self-concept, they are more likely to engage in innovation to maintain and reinforce that identity (Beijaard et al., 2004). For teachers, PI is closely tied to educational mission, student development, and social responsibility (Liu and Geertshuis, 2016). As GenAI reshapes instructional practices and role boundaries, teachers with stronger PI may view the responsible use of AI-generated content and human–AI collaborative teaching as part of their professional responsibility and therefore engage more actively in innovative practice (Avidov-Ungar and Forkosh-Baruch, 2018). AI-TIB, as a context-specific form of innovation, similarly depends on identity commitment and role responsibility. PI may not only relate directly to innovative behavior but may also help explain how competence is connected to practice, especially when teachers must continuously adapt to the pedagogical and professional challenges introduced by GenAI. Thus, PI may connect AI-TPACK to AI-TIB. Accordingly, we propose:

*H8*: Each dimension of AI-TPACK is positively associated with college teachers’ PI.

*H9*: PI is positively associated with college teachers’ AI-TIB.

*H10*: PI mediates the associations between AI-TPACK dimensions and AI-TIB.

### 2.6 The mediating role of AI literacy

In educational contexts, the deep integration of GenAI into teaching has made AIL an increasingly important component of teachers’ professional competence. Prior studies grounded in the TPACK framework report significant associations between teachers’ technology integration knowledge and the development of AIL (Al-Abdullatif, 2024; Pehlevan and Ünal, 2024; Wilton et al., 2024). TPACK emphasizes the dynamic integration of technology, pedagogy, and content (Mishra and Koehler, 2006), which enables teachers to move beyond basic tool operation and align technology with instructional goals. Empirical evidence suggests that higher TPACK relates to stronger technological understanding and application in GenAI-supported environments (Celik, 2023). In the era of intelligent education, GenAI’s generative, adaptive, and algorithm-driven features require expanded integration competence, including the ability to evaluate AI-generated content, understand algorithmic recommendations, manage ethical risks, and effectively coordinate human–AI collaboration within instructional settings (Caspari-Sadeghi, 2026). AI-TPACK, as an extension of TPACK, highlights this deeper integration in AI contexts (Ning et al., 2024). Although research has not directly examined the association between AI-TPACK and AIL, established links between TPACK and AIL suggest that AI-TPACK may play a stronger role in AI settings. For example, Wilton et al. (2024) noted that extending TPACK to AI contexts supports the development of educators’ AIL. Taken together, technology integration knowledge provides a cognitive foundation for AIL. In GenAI-supported environments, this integrative capacity is reflected in AI-TPACK, which enables teachers to translate AI capabilities into pedagogically meaningful instructional practices.

Prior research indicates that AIL relates to individuals’ innovative behavior in AI contexts (Ding et al., 2025; Wang W. B. et al., 2026). AIL involves not only understanding AI principles but also the ability to critically evaluate AI-generated outputs, creatively integrate AI-supported resources, and solve instructional problems in specific teaching situations. Studies show that individuals with higher AIL demonstrate stronger technological judgment and operational confidence, which increases their engagement in AI-related practice (Ji et al., 2025). For example, Wang et al. (2023) found that teachers with higher AIL used generative AI tools more effectively to generate ideas and solve problems, thereby supporting innovative behavior. From a competence perspective, innovation depends not only on access to technology but also on accurate evaluation of technological value, contextual integration skills, and risk management capacity. Teachers with stronger AIL can assess the applicability, limitations, and reliability of AI-generated content and adjust AI-supported resources according to instructional goals (Wang and Tan, 2025). In addition, the ethical awareness component of AIL helps teachers identify and manage potential risks, reducing uncertainty-related anxiety (Mailizar et al., 2022). This sense of understanding and control over AI environments provides an important foundation for innovative practice. Although prior research has mainly focused on the association between AIL and technology adoption or acceptance, the competence demands of innovation suggest a broader role for AIL in GenAI-supported teaching. In GenAI-supported teaching contexts, AIL may not only relate to technology adoption but also to teachers’ ability and sustained engagement in transforming AI-generated outputs and human–AI collaborative processes into innovative instructional practice. Thus, AIL is likely positively associated with college teachers’ AI-TIB. Furthermore, AI-TPACK represents an integrative professional knowledge base in AI contexts, whereas AIL reflects the practical capacity to apply this knowledge. Knowledge structures may support innovation only when they translate into contextual judgment and effective technology integration. Therefore, AIL may mediate the association between AI-TPACK and AI-TIB. Accordingly, we propose:

*H11*: Each dimension of AI-TPACK is positively associated with college teachers’ AIL.

*H12*: AIL is positively associated with college teachers’ AI-TIB.

*H13*: AIL mediates the associations between AI-TPACK dimensions and AI-TIB.

**Figure 2:**
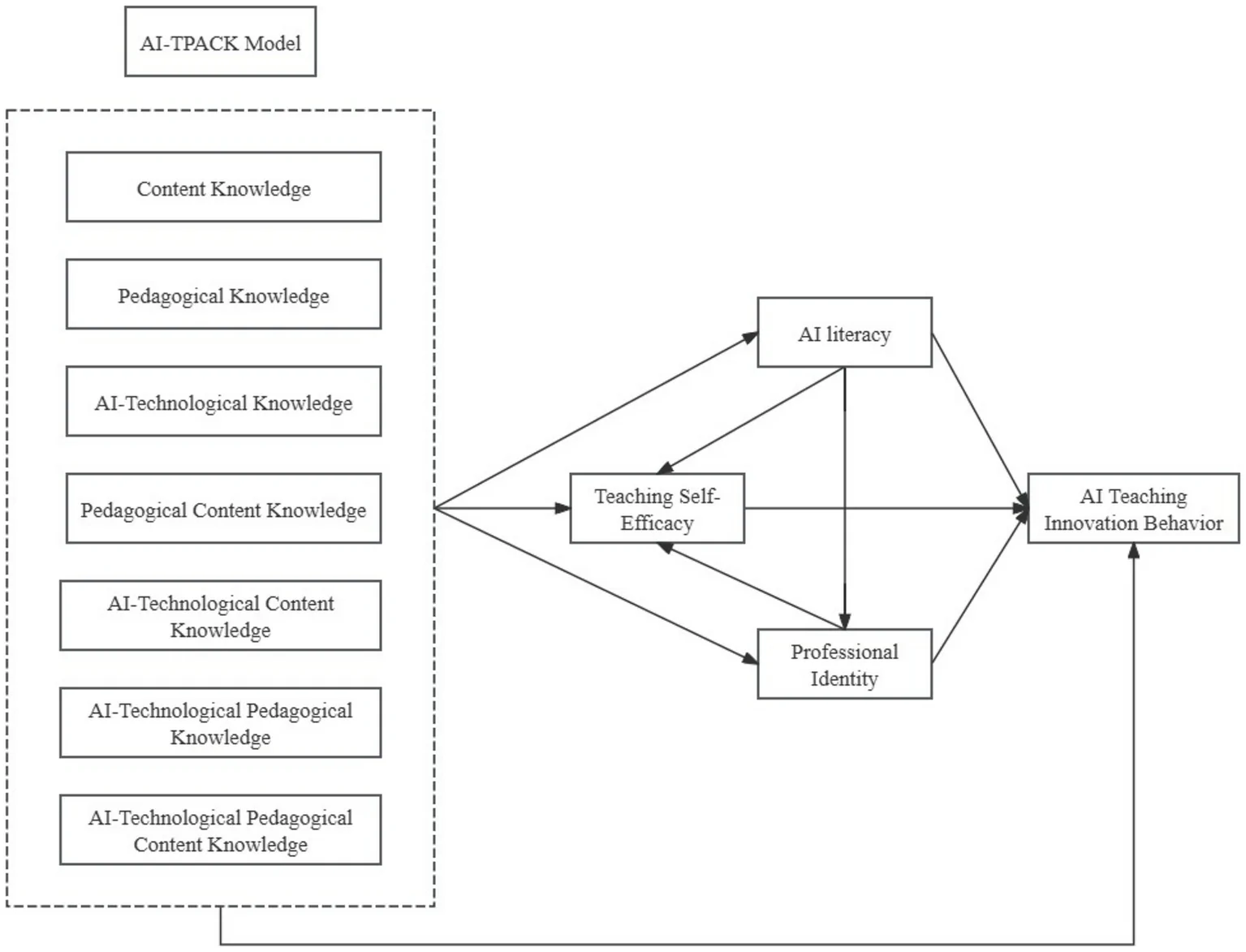
Research model.

Based on these hypotheses, the study presents the hypothesized model (Figure 2). Although the hypotheses are presented at the dimension level, they are organized under a common theoretical framework. This approach was adopted because prior research suggests that different AI-TPACK dimensions may contribute differently to teachers’ AI-related outcomes. Examining the dimensions separately allows the study to explore which specific knowledge domains show stronger associations with AI-TIB and relevant psychological factors.

## 3 Method

### 3.1 Sample source and data collection

Faculty members from the cooperating universities helped distribute the questionnaires through the online survey platform Wenjuanxing between February and March 2026. A convenience sampling approach was used. Participation was entirely voluntary, and all respondents provided informed consent prior to taking part. The sample was drawn from eight universities selected based on three criteria: (1) geographical diversity, covering eastern, central, and western regions of China; (2) institutional diversity, including public undergraduate universities, one private undergraduate university, and one higher vocational college; and (3) active engagement in AI-related educational development. Specifically, all participating institutions had established AI-related initiatives, such as AI curriculum development, smart course construction, faculty AI training programs, or institutional plans promoting AI-supported teaching innovation. The participating universities included institutions from Shanxi, Shaanxi, Shandong, Yunnan, Hebei, Henan, and other regions, representing different stages of higher education development in China. Although the sample was obtained through convenience sampling, the inclusion of institutions with diverse regional and organizational characteristics was intended to enhance contextual coverage and improve the transferability of the findings. The target population of this study was college teachers with current teaching responsibilities; therefore, in addition to lecturers, associate professors, and professors, the sample also included teaching assistants and other junior instructional staff who were actively involved in course-related teaching activities. While this sampling method may introduce self-selection bias and limit the generalizability of findings, it was considered the most feasible option given the practical constraints and available resources.

According to Kline (2018) recommendation, each item in the questionnaire should receive responses from at least 10 participants. The instrument comprised 109 items, and assuming a potential 20% attrition rate, the target sample was calculated to be 1,272 participants (106 × 10 + 20% × 106 × 10). Of the 1,350 questionnaires distributed, 1,100 were returned. During data screening, 202 responses were excluded because either more than 20% of the items were left unanswered, or over 80% of the items were consistently answered with extreme options (i.e., strongly agree or strongly disagree). Such patterns could cause substantial bias, commonly referred to as floor or ceiling effects, potentially undermining the accuracy of analysis (Wang et al., 2012). We ultimately retained 898 valid questionnaires for analysis. Although the final analytic sample was smaller than the conservative planning target, the 898 valid cases represented 81.64% of all returned questionnaires and still provided an adequate sample size for the analyses conducted in this study.

We summarize the demographic profile of the participants in Table 1: 312 males (34.74%) and 586 females (65.26%); 27.8% of participants (250) were 25 years or younger; the majority held undergraduate degrees (43.43%, 390 participants); teaching assistants constituted the largest position category (34.08%, 306 participants); most participants had 1–3 years of teaching experience (29.84%, 268 participants); 43.54% (391) were from humanities and social sciences; and 26.28% (236 participants) reported using AI tools multiple times daily. The relatively high proportions of younger respondents, bachelor’s-degree holders, and teaching assistants reflect the inclusion of early-career instructional personnel in Chinese higher education. In some university settings, such staff may undertake tutorials, laboratory instruction, practice-based teaching, and other course-support responsibilities while still being at an early career stage or before completing higher academic degrees. Regarding AI utilization frequency, prior AI use was not established as an inclusion criterion in this study. The small proportion of teachers who reported never using AI tools were retained because they were recruited from universities where AI-related educational initiatives and institutional support were actively promoted. No personally identifiable information was collected or disclosed.

**Table 1:** Demographic characteristics.

Variables	Options	*N*	Percentage (%)
Gender	Man	312	34.74%
	Woman	586	65.26%
Age	Aged 25 and under	250	27.84%
	25–35 years old	194	21.60%
	35–45 years old	243	27.06%
	45–55 years old	138	15.37%
	Aged 55 and above	73	8.13%
Academic Qualification	Ph. D.	161	17.93%
	Master	347	38.64%
	Bachelor	390	43.43%
Academic Rank/Position	Teaching Assistant	306	34.08%
	Lecturer	215	23.94%
	Associate Professor	257	28.62%
	Professor	120	13.36%
Teaching Tenure	1–3 years	268	29.84%
	4–6 years	172	19.15%
	7–10 years	113	12.58%
	11-15 years	136	15.14%
	16 years and above	209	23.27%
Discipline Classification	Humanities and Social Sciences	391	43.54%
	Science and Engineering	251	27.95%
	Medical and Health Sciences	186	20.71%
	Arts and Physical Education	70	7.80%
AI Utilization Frequency	Several times a day	236	26.28%
	Once a day	153	17.04%
	Several times a month	373	41.54%
	Rarely used	109	12.14%
	Never used	27	3.01%

### 3.2 Measurement instruments

We used established and validated scales for all constructs. In addition to evaluating reliability and validity in the present sample, we also considered psychometric evidence reported by the original developers and subsequent validation studies, particularly those involving Chinese educational populations. Except for demographic variables, all measures used a five-point Likert-type format. All English-language instruments were translated and back-translated for use in the Chinese context, and bilingual experts in psychology, educational technology, teacher education, and foreign language education reviewed the wording to ensure semantic equivalence and contextual appropriateness. For scales involving AI-related teaching practices, items were checked and, where necessary, contextually adapted to explicitly refer to AI-supported teaching. The translated and adapted items were further reviewed by experts for relevance and clarity, and a pilot test with 50 participants indicated that the items were clearly understood and appropriate for the present study.

#### 3.2.1 AI-TPACK

We measured AI-TPACK using the scale developed by Ning et al. (2024). The instrument assesses teachers’ professional cognition in technology-integrated instruction across seven dimensions: CK, AI-TK, PK, AI-TPK, AI-TCK, PCK, and AI-TPACK. The scale contains 42 items (e.g., “I can keep up with important developments in AI technology”). All items were rated on a five-point Likert scale ranging from 1 (strongly disagree) to 5 (strongly agree), with higher scores indicating higher levels of AI-related professional cognition in AI-supported teaching. In this study, the Cronbach’s *α* coefficients for the seven dimensions were 0.935, 0.941, 0.946, 0.948, 0.944, 0.935, and 0.943, respectively, indicating good internal consistency.

#### 3.2.2 Teaching self-efficacy

The TSE was assessed using the 12-item short-form Teacher Sense of Efficacy Scale (TSES-SF) developed by Tschannen-Moran and Hoy (2001), which measures teachers’ beliefs in their instructional capabilities. The 12 items are grouped into three sub-dimensions: Instructional Strategies, Classroom Management, and Student Engagement (e.g., “I can implement a variety of assessment strategies”). The scale used a 5-point response format ranging from 1 (no confidence) to 5 (complete confidence), with higher scores indicating higher TSE. The scale has been widely validated among Chinese teachers, demonstrating good reliability and validity (Li, 2023; Long and Xia, 2025; Shao et al., 2025). Following factor loading assessment, items with relatively low loadings were removed, and eight items were retained for subsequent analyses. The overall Cronbach’s α was 0.937, indicating satisfactory internal consistency.

#### 3.2.3 Professional identity

This study employed the Professional Identity Five-Factor Scale (PIFFS) developed by Tan et al. (2017) to measure university teachers’ professional identity. The scale consists of five dimensions: knowledge about professional practices, experience with the profession, having the professional as a role model, professional self-efficacy, and preference for a particular profession. The scale comprises 25 items, and responses were rated on a five-point Likert scale ranging from 1 (strongly disagree) to 5 (strongly agree). Following factor loading assessment, items with relatively low loadings were removed, and 19 items were retained for subsequent analyses. Higher scores indicate stronger professional identity. In this study, the overall Cronbach’s *α* was 0.934, indicating satisfactory internal consistency.

#### 3.2.4 AI literacy

The AIL was measured using the scale developed by Ng et al. (2021), which includes four dimensions: Knowing and Understanding AI, Applying AI, Evaluating AI Application, and AI Ethics, with a total of 20 items (e.g., “I can distinguish between smart educational devices and non-smart educational devices”). Responses were rated on a five-point Likert scale ranging from 1 (you have no AI literacy at all) to 5 (you dominate AI completely), with higher scores indicating higher levels of AIL. The scale has demonstrated good reliability and validity among Chinese teachers (Yang and Li, 2026; Zhao et al., 2022). In this study, the overall Cronbach’s α for the AIL scale was 0.932.

#### 3.2.5 AI teaching innovation behavior

To measure teachers’ innovative behavior in AI-supported instructional contexts, this study adopted the Innovation Behavior Scale originally developed by Scandura et al. (1986) and revised by Byun et al. (2012) and Kim and Hong (2013). Because the original scale was designed to assess general innovative behavior, the original item wording was retained in this study. To anchor participants’ responses to AI-related instructional practice, a brief contextual instruction was provided before the scale. Respondents were asked to answer the items with reference to their teaching in a GenAI-supported environment. In addition, expert review supported the appropriateness of using the scale in this context and the clarity of the scenario-based instruction for capturing AI teaching innovation behavior. The scale comprising seven items (e.g., “I have proposed and attempted to implement new ideas to solve complex or challenging problems”). Items were rated on a five-point Likert scale ranging from 1 (strongly disagree) to 5 (strongly agree), with higher scores indicating higher levels of AI teaching innovation behavior. The scale has shown good reliability and validity among Chinese teachers (Lin and Shin, 2021). In this study, the overall Cronbach’s α for the AI-TIB scale was 0.933.

### 3.3 Data analysis

This study analyzed the data using SPSS 27.0 and SmartPLS 4.0. Following a two-stage PLS-SEM procedure (Hair et al., 2019), the measurement model was first evaluated for internal consistency reliability, convergent validity (Fornell and Larcker, 1981; Hair et al., 2019), and discriminant validity using the heterotrait–monotrait ratio (HTMT) (Henseler et al., 2015). Next, the structural model was examined using bootstrapping with 5,000 resamples to estimate path coefficients and 95% confidence intervals; effects, including indirect effects, were considered significant when the confidence interval did not include zero (Hair et al., 2019; Nitzl et al., 2016). PLS-SEM was selected because the proposed model was relatively complex, involving seven AI-TPACK dimensions, three mediators, multiple direct and indirect paths, and a multi-group analysis. In addition, the study aims to test theoretical relationships among latent constructs rather than confirm a well-established covariance structure, making PLS-SEM appropriate for examining complex path relationships in this context. Explanatory power and predictive relevance of the structural model were evaluated using the coefficient of determination (*R*^2^) and predictive relevance (*Q*^2^), respectively. In addition, overall model fit was descriptively examined using SRMR and NFI within the PLS-SEM framework. All measurement and hypothesis testing procedures were conducted consistently within the PLS-SEM framework.

## 4 Results

### 4.1 Descriptive statistics

This study conducted correlation analyses on CK, AI-TK, PK, AI-TPK, PCK, AI-TCK, AI-TPACK, TSE, PI, AIL, and AI-TIB. Table 2 presents the sample size (N), mean (M), standard deviation (SD), kurtosis (Kur), and skewness (Sk) for each variable. The absolute values of skewness were below 2, and the absolute values of kurtosis were below 7, indicating that the variables approximately followed a normal distribution.

**Table 2:** Means, standard deviations, Sk, Kur and sample size.

Variables	*N*	*M*	SD	Sk	Kur
PK	898	18.26	3.628	−0.269	0.034
CK	898	18.10	3.662	0.049	0.084
AI-TK	898	19.74	3.480	−0.274	−0.168
PCK	898	17.77	3.098	0.346	0.036
AI-TCK	898	16.67	3.654	0.186	−0.006
AI-TPK	898	17.93	3.697	−0.057	0.086
AI-TPACK	898	17.52	3.141	0.328	0.026
AIL	898	60.45	10.997	−0.207	0.011
TSE	898	25.88	4.662	0.122	−0.025
PI	898	55.56	11.557	−0.242	0.109
AI-TIB	898	21.45	4.387	0.040	−0.050

CK, content knowledge; AI-TK, AI-technological knowledge; PK, pedagogical knowledge; PCK, pedagogical-content knowledge; AI-TCK, AI-technological-content knowledge; AI-TPK, AI-technological-pedagogical knowledge; AI-TPACK, AI-Technological Pedagogical Content Knowledge; AIL, AI literacy; TSE, teaching self-efficacy; PI, professional identity; AI-TIB, AI teaching innovation behavior.

### 4.2 Common method bias

Common method bias (CMB) was assessed using both Harman’s single-factor test and the common latent factor (CLF) approach. For Harman’s single-factor test, all measurement items were entered into an unrotated exploratory factor analysis. The first unrotated factor accounted for 33.31% of the total variance, which was below the recommended threshold of 40% (Kock et al., 2021), suggesting that CMB was unlikely to be a serious concern. To further examine potential method bias, a CLF model was estimated by introducing a CLF that loaded on all observed indicators. The chi-square difference between the baseline model and the CLF model was not statistically significant (Δ*χ*^2^ = 3.583, Δdf = 1, *p* > 0.05), indicating that the inclusion of a CLF did not significantly improve model fit. Therefore, the results suggest that CMB did not substantially affect the findings of this study.

### 4.3 PLS-SEM results

#### 4.3.1 Measurement model assessment

Following established PLS-SEM procedures (Hair et al., 2022), the measurement model was assessed in terms of internal consistency reliability, convergent validity, and discriminant validity. As shown in Table 3, all composite reliability (CR) values exceeded 0.70, and all average variance extracted (AVE) values exceeded 0.50, indicating satisfactory internal consistency reliability and convergent validity. Although several Cronbach’s alpha and CR values were relatively high, which may raise concerns about possible item redundancy, the retained items were drawn from established scales and were intended to capture conceptually distinct facets of each construct rather than repetitive wording of the same content. Specifically, the measures covered multiple theoretically relevant dimensions, such as the seven dimensions of AI-TPACK, the three dimensions of TSE, the five dimensions of PI, and the four dimensions of AIL, thereby suggesting that the measures provided acceptable content coverage in addition to high internal consistency.

**Table 3:** Reliability and validity.

Constructs	Cronbach’s alpha	CR	AVE
CK	0.935	0.949	0.755
AI-TK	0.941	0.953	0.771
PK	0.946	0.957	0.787
AI-TPK	0.948	0.958	0.793
AI-TCK	0.944	0.956	0.782
PCK	0.935	0.949	0.755
AI-TPACK	0.943	0.955	0.779
TSE	0.937	0.934	0.768
PI	0.934	0.935	0.781
AIL	0.932	0.934	0.751
AI-TIB	0.933	0.939	0.818

The above indicators demonstrate that the measurement tools exhibit good reliability and validity, making them suitable for further modeling analysis. CK, Content knowledge; AI-TK, AI-technological knowledge; PK, pedagogical knowledge; PCK, pedagogical-content knowledge; AI-TCK, AI-technological-content knowledge; AI-TPK, AI-technological-pedagogical knowledge; AI-TPACK, AI-Technological Pedagogical Content Knowledge; AIL, AI literacy; TSE, teaching self-efficacy; PI, professional identity; AI-TIB, AI teaching innovation behavior.

Discriminant validity was further assessed using the HTMT and the Fornell–Larcker criterion. As shown in Table 4, all HTMT values were below the recommended threshold of 0.85. In addition, Table 5 shows that the square root of each construct’s AVE exceeded its correlations with other constructs, thus supporting discriminant validity. It is worth noting, however, that several correlations among the TPACK-related dimensions were very small, with a few trivially negative coefficients. Although this pattern is somewhat unusual for theoretically related constructs, these coefficients were close to zero rather than indicating substantive inverse relationships. One possible explanation is that the AI-TPACK dimensions represented relatively differentiated knowledge domains and may have developed unevenly across participants from different disciplines, institutions, and career stages. Therefore, while the HTMT, AVE, CR, and factor-loading results supported the adequacy of the measurement model, the structural coherence among some TPACK dimensions should be interpreted with caution and examined further in future research. Overall, the measurement model demonstrated acceptable reliability, convergent validity, and discriminant validity.

**Table 4:** Discriminant validity (HTMT criterion).

Constructs	AIL	AI-TPACK	CK	AI-TIB	AI-TCK	AI-TPK	AI-TK	PCK	TSE	PK	PI
AIL											
AI-TPACK	0.277										
CK	0.174	0.042									
AI-TIB	0.685	0.232	0.225								
AI-TCK	0.166	0.027	0.030	0.243							
AI-TPK	0.183	0.047	0.029	0.213	0.051						
AI-TK	0.210	0.048	0.023	0.158	0.037	0.036					
PCK	0.168	0.060	0.106	0.266	0.058	0.051	0.033				
TSE	0.591	0.223	0.169	0.675	0.215	0.193	0.140	0.216			
PK	0.260	0.035	0.043	0.259	0.040	0.038	0.029	0.045	0.257		
PI	0.555	0.253	0.152	0.650	0.170	0.186	0.181	0.283	0.586	0.221	

CK, content knowledge; AI-TK, AI-technological knowledge; PK, pedagogical knowledge; PCK, pedagogical-content knowledge; AI-TCK, AI-technological-content knowledge; AI-TPK, AI-technological-pedagogical knowledge; AI-TPACK, AI-Technological Pedagogical Content Knowledge; AIL, AI literacy; TSE, teaching self-efficacy; PI, professional identity; AI-TIB, AI teaching innovation behavior.

**Table 5:** Discriminant validity (Fornell–Larcker criterion).

Constructs	AIL	AI-TPACK	CK	AI-TIB	AI-TCK	AI-TPK	AI-TK	PCK	TSE	PK	PI
AIL	0.866										
AI-TPACK	0.267	0.867									
CK	0.175	0.035	0.876								
AI-TIB	0.669	0.212	0.226	0.906							
AI-TCK	0.165	−0.024	0.007	0.236	0.889						
AI-TPK	0.182	0.054	−0.002	0.209	−0.031	0.891					
AI-TK	0.205	−0.044	−0.016	0.157	0.010	0.027	0.887				
PCK	0.173	−0.066	0.088	0.259	0.056	−0.025	0.033	0.864			
TSE	0.575	0.212	0.172	0.657	0.207	0.202	0.140	0.205	0.876		
PK	0.247	0.000	−0.022	0.240	−0.030	−0.034	0.002	0.021	0.249	0.882	
PI	0.552	0.247	0.146	0.642	0.161	0.186	0.169	0.281	0.579	0.227	0.885

According to the Fornell–Larcker criterion, to assess discriminant validity, the correlations between constructs should be less than the square root of the average variance extracted (AVE) (the diagonal values represent the square roots of AVE). CK, content knowledge; AI-TK, AI-technological knowledge; PK, pedagogical knowledge; PCK, pedagogical-content knowledge; AI-TCK, AI-technological-content knowledge; AI-TPK, AI-technological-pedagogical knowledge; AI-TPACK, AI-Technological Pedagogical Content Knowledge; AIL, AI literacy; TSE, teaching self-efficacy; PI, professional identity; AI-TIB, AI teaching innovation behavior.

#### 4.3.2 Structural model assessment

For the structural model analysis in this study, several indicators were used, including collinearity diagnostics, path coefficients, and the coefficient of determination. These indicators help assess the model’s reliability and explanatory power. Collinearity diagnostics were conducted to check for potential multicollinearity issues (Leguina, 2015). According to the common rule of thumb, all variables should have variance inflation factor (VIF) values below the threshold of 3, indicating no serious multicollinearity problems. As shown in Table 6, the VIF values of all variables ranged from 1.003 to 1.863, suggesting that multicollinearity was not a concern.

**Table 6:** Multicollinearity test.

Constructs	AIL	AI-TPACK	CK	AI-TIB	AI-TCK	AI-TPK	AI-TK	PCK	TSE	PK	PI
AIL				1.816					1.649	1.421	
AI-TPACK	1.011			1.166					1.161	1.118	
CK	1.010			1.062					1.053	1.047	
AI-TIB											
AI-TCK	1.006			1.087					1.064	1.048	
AI-TPK	1.006			1.094					1.077	1.052	
AI-TK	1.004			1.079					1.078	1.065	
PCK	1.018			1.136					1.130	1.054	
TSE				1.862							
PK	1.003			1.153					1.130	1.098	
PI				1.838					1.661		

AIL, AI literacy; AI-TPACK, AI-Technological Pedagogical Content Knowledge; CK, content knowledge; AI-TIB, AI teaching innovation behavior; AI-TCK, AI-technological-content knowledge; AI-TPK, AI-technological-pedagogical knowledge; AI-TK, AI-technological knowledge; PCK, pedagogical-content knowledge; TSE, teaching self-efficacy; PK, pedagogical knowledge; PI, professional identity.

#### 4.3.3 Path hypothesis testing

This study assessed the magnitude and significance of the model’s path coefficients through a PLS bootstrapping procedure with 5,000 subsamples, while controlling for age, educational level, and professional title. Given the relatively large sample size (*N* = 898), effect sizes (*f*^2^) were also examined alongside statistical significance. The corresponding *f*^2^ values are reported in Supplementary Table S1. Table 7 presents the PLS results. In structural modeling, significance testing is used to examine the relationships between exogenous and endogenous variables. The path analysis results indicate that AI-TPACK dimensions were significantly and positively associated with college teachers’ PI and AIL, supporting H8 and H11. Additionally, TSE, PI, and AIL were significantly associated with teachers’ AI-TIB; PI and AIL were also associated with TSE, and AIL was associated with PI. Therefore, H2–H4, H6, H9, and H12 were supported. However, except for AI-TK and AI-TPACK, the remaining dimensions of AI-TPACK were significantly associated with AI-TIB, resulting in partial support for H1. AI-TK was not associated with TSE, whereas the other dimensions of AI-TPACK showed significant associations with TSE, leading to partial support for H5. The *f*^2^ results suggested that several statistically significant paths were small in magnitude and should therefore be interpreted cautiously in terms of practical relevance.

**Table 7:** Path hypothesis testing.

Hypothesis	Original Sample (O)	2.5%	97.5%	*t*	*p*	Results
CK → AI-TIB	0.085	0.045	0.125	4.167	<0.001	Supported
AI-TK → AI-TIB	0.012	−0.027	0.055	0.585	0.558	Not Supported
PK → AI-TIB	0.048	0.003	0.096	2.046	0.041	Supported
AI-TPK → AI-TIB	0.060	0.019	0.100	2.849	0.004	Supported
AI-TCK → AI-TIB	0.091	0.046	0.135	3.979	<0.001	Supported
PCK → AI-TIB	0.072	0.029	0.116	3.232	<0.001	Supported
AI-TPACK → AI-TIB	0.014	−0.031	0.058	0.610	0.542	Not Supported
CK → TSE	0.069	0.022	0.118	2.820	0.005	Supported
AI-TK → TSE	0.024	−0.023	0.072	0.971	0.332	Not Supported
PK → TSE	0.111	0.060	0.160	4.313	<0.001	Supported
AI-TPK → TSE	0.095	0.050	0.137	4.260	<0.001	Supported
AI-TCK → TSE	0.111	0.062	0.160	4.446	<0.001	Supported
PCK → TSE	0.057	0.003	0.112	2.023	0.043	Supported
AI-TPACK → TSE	0.055	0.005	0.105	2.158	0.031	Supported
CK → PI	0.060	0.010	0.111	2.378	0.017	Supported
AI-TK → PI	0.089	0.040	0.141	3.468	0.001	Supported
PK → PI	0.139	0.084	0.193	5.005	<0.001	Supported
AI-TPK → PI	0.121	0.070	0.173	4.572	<0.001	Supported
AI-TCK → PI	0.098	0.047	0.149	3.760	<0.001	Supported
PCK → PI	0.214	0.165	0.266	8.236	<0.001	Supported
AI-TPACK → PI	0.160	0.105	0.215	5.826	<0.001	Supported
CK → AIL	0.160	0.102	0.217	5.497	<0.001	Supported
AI-TK → AIL	0.207	0.150	0.265	7.120	<0.001	Supported
PK → AIL	0.258	0.204	0.312	9.325	<0.001	Supported
AI-TPK → AIL	0.180	0.125	0.235	6.366	<0.001	Supported
AI-TCK → AIL	0.173	0.122	0.228	6.473	<0.001	Supported
PCK → AIL	0.159	0.106	0.210	5.895	<0.001	Supported
AI-TPACK → AIL	0.275	0.225	0.325	10.663	<0.001	Supported
TSE → AI-TIB	0.258	0.198	0.317	8.357	<0.001	Supported
PI → AI-TIB	0.244	0.188	0.296	8.804	<0.001	Supported
PI → TSE	0.309	0.248	0.368	10.071	<0.001	Supported
AIL → TSE	0.300	0.238	0.361	9.598	<0.001	Supported
AIL → PI	0.370	0.314	0.424	13.086	<0.001	Supported
AIL → AI-TIB	0.315	0.261	0.369	11.296	<0.001	Supported

The hypothesized model reported the original sample, confidence intervals, *t*-statistics, and *p*-values, with *t*-values expected to exceed 1.96 and *p*-values expected to be below 0.05. CK, content knowledge; AI-TK, AI-technological knowledge; PK, pedagogical knowledge; AI-TPK, AI-technological-pedagogical knowledge; AI-TCK, AI-technological-content knowledge; PCK, pedagogical-content knowledge; AI-TPACK, AI-Technological Pedagogical Content Knowledge; AIL, AI literacy; TSE, teaching self-efficacy; PI, professional identity; AI-TIB, AI teaching innovation behavior.

The *R*^2^ and *Q*^2^ values of the endogenous constructs represent the model’s explanatory power and predictive relevance, respectively (Hair et al., 2022). As shown in Table 8, the *R*^2^ values indicate acceptable explanatory power. Specifically, the predictors explained 62.50% of the variance in AI-TIB, 29.60% of the variance in AIL, 46.00% of the variance in TSE, and 39.80% of the variance in PI. Moreover, all *Q*^2^ values exceeded zero (see Table 8), indicating that the empirical model had moderate predictive relevance. The SRMR was 0.027 and the NFI was 0.898, both of which suggested a good model fit.

**Table 8:** Explanatory power and predictive relevance.

Constructs	*R* ^2^	*R* ^2^_adjust_	*Q* ^2^	Model fit
AI-TIB	0.625	0.621	0.509	SRMR: 0.027 NFI: 0.898
AIL	0.296	0.291	0.220	
TSE	0.460	0.455	0.349	
PI	0.398	0.393	0.309	

“*R*^2^” represents the model’s explanatory power, “*Q*^2^” indicates predictive relevance, “SRMR” stands for standardized root mean square residual, and “NFI” denotes the normed fit index.

### 4.4 Mediation analysis

This study conducted mediation analysis using the bootstrap method in PLS-SEM to examine whether TSE, PI, and AIL mediate the relationships between AI-TPACK and AI-TIB. The significance of mediation effects was evaluated primarily based on whether the bootstrapped 95% confidence interval for the indirect effect excluded zero, while the direct effects were examined simultaneously to identify the type and extent of mediation.

As shown in Table 9, different types of mediation were observed across the tested paths. For most AI-TPACK dimensions, the indirect effects through PI and AIL were significant while the corresponding direct effects on AI-TIB also remained significant, indicating partial mediation. By contrast, for AI-TK and AI-TPACK, the direct effects on AI-TIB were not significant, whereas some indirect effects remained significant, indicating indirect-only mediation rather than partial mediation. Specifically, AI-TK showed indirect-only mediation through PI and AIL, whereas AI-TPACK showed indirect-only mediation through TSE, PI, and AIL. However, the indirect effect through TSE was not supported for the path AI-TK → TSE → AI-TIB. Thus, the only unsupported indirect path was AI-TK → TSE → AI-TIB, and H7 was therefore partially supported. By contrast, all indirect paths through PI were supported, supporting H10, and all indirect paths through AIL were also supported, supporting H13. It should also be noted that the indirect effect of PCK → TSE → AI-TIB was retained as supported because its bootstrapped 95% confidence interval excluded zero, although its *p*-value was marginal (*p* = 0.054). In detail, within the relationship between AI-TPACK and AI-TIB, enhancing TSE, strengthening PI, and cultivating AIL can collectively promote AI-TIB. However, although most indirect effects were statistically supported, their magnitudes were generally small and should therefore be interpreted cautiously in terms of practical significance.

**Table 9:** Mediation analysis results.

Relationship	Indirect effect	2.5%	97.5%	*t*	*p*	Direct effect	*t*	*p*	Hypotheses
The mediating role of TSE									
CK → TSE → AI-TIB	0.018	0.005	0.032	2.682	0.007	0.085	4.167	<0.001	Supported
AI-TK → TSE → AI-TIB	0.006	−0.006	0.019	0.964	0.335	0.012	0.585	0.558	Not Supported
PK → TSE → AI-TIB	0.029	0.015	0.044	3.739	<0.001	0.048	2.046	0.041	Supported
AI-TPK → TSE → AI-TIB	0.024	0.013	0.037	3.922	<0.001	0.060	2.849	0.004	Supported
AI-TCK → TSE → AI-TIB	0.029	0.016	0.042	4.238	<0.001	0.091	3.979	<0.001	Supported
PCK → TSE → AI-TIB	0.015	0.001	0.030	1.927	0.054	0.072	3.232	<0.001	Supported
AI-TPACK →TSE → AI-TIB	0.014	0.001	0.028	2.052	0.040	0.014	0.610	0.542	Supported
The mediating role of PI									
CK → PI → AI-TIB	0.015	0.003	0.028	2.318	0.021	0.085	4.167	<0.001	Supported
AI-TK → PI → AI-TIB	0.022	0.009	0.037	3.118	0.002	0.012	0.585	0.558	Supported
PK → PI → AI-TIB	0.034	0.019	0.051	4.182	<0.001	0.048	2.046	0.041	Supported
AI-TPK → PI → AI-TIB	0.030	0.016	0.045	3.887	<0.001	0.060	2.849	0.004	Supported
AI-TCK → PI → AI-TIB	0.024	0.011	0.038	3.448	0.001	0.091	3.979	<0.001	Supported
PCK → PI → AI-TIB	0.052	0.035	0.072	5.549	<0.001	0.072	3.232	<0.001	Supported
AI-TPACK → PI → AI-TIB	0.039	0.024	0.055	4.992	<0.001	0.014	0.610	0.542	Supported
The mediating role of AIL									
CK → AIL → AI-TIB	0.050	0.031	0.071	4.978	<0.001	0.085	4.167	<0.001	Supported
AI-TK → AIL → AI-TIB	0.065	0.045	0.086	6.198	<0.001	0.012	0.585	0.558	Supported
PK → AIL → AI-TIB	0.081	0.060	0.104	7.382	<0.001	0.048	2.046	0.041	Supported
AI-TPK → AIL → AI-TIB	0.057	0.038	0.078	5.525	<0.001	0.060	2.849	0.004	Supported
AI-TCK → AIL → AI-TIB	0.055	0.037	0.075	5.613	<0.001	0.091	3.979	<0.001	Supported
PCK → AIL → AI-TIB	0.050	0.032	0.069	5.159	<0.001	0.072	3.232	<0.001	Supported
AI-TPACK → AIL → AI-TIB	0.086	0.065	0.110	7.580	<0.001	0.014	0.610	0.542	Supported

“*t*” denotes two-tailed *t*-test value, “*p*” indicates significance level. CK, content knowledge; AI-TK, AI-technological knowledge; PK, pedagogical knowledge; AI-TPK, AI-technological-pedagogical knowledge; AI-TCK, AI-technological-content knowledge; PCK, pedagogical-content knowledge; AI-TPACK, AI-Technological Pedagogical Content Knowledge; AIL, AI literacy; TSE, teaching self-efficacy; PI, professional identity; AI-TIB, AI teaching innovation behavior.

### 4.5 Multi-group analysis

To explore the differential effects of AI tool usage frequency on college teachers’ AI-TIB, this study conducted a multi-group analysis (MGA). MGA primarily examines the differences in path coefficients between groups to test inter-group differences (Hair et al., 2017). Before conducting MGA, we performed a Measurement Invariance of Composites (MICOM) test to identify the sources of differences in the structural relationships among latent variables. MGA is appropriate when the differences between groups arise from varying interpretations or understandings of the measured phenomena rather than from differences in structural relationships (Henseler et al., 2016). The following sections describe the MICOM and MGA procedures and results.

#### 4.5.1 Measurement invariance of composites (MICOM)

During the analysis, considering the detailed classification of AI tool usage frequency, and to align with the requirements of MGA in Smart-PLS, we categorized participants based on their usage patterns. Daily users (once or multiple times per day) were assigned to the “high-frequency group,” while those using AI tools several times per month, rarely, or never were assigned to the “low-frequency group.” This classification provides a clearer basis for analyzing user activity and its association with the study outcomes. The high-frequency group included 389 participants, and the low-frequency group included 509 participants.

To assess whether the constructs of the measurement instruments were comparable and to evaluate the compositional invariance of the model, we conducted 1,000 permutation tests. According to Henseler et al. (2016), compositional invariance is established when the original correlation is equal to or greater than the 5% quantile of the permutation distribution; the permutation p value can also be used as supplementary evidence, with values above 0.05 indicating that compositional invariance cannot be rejected. As shown in Table 10, the original correlations for all constructs were equal to or greater than the corresponding 5% quantiles. Although the AI-TK value was comparatively lower than those of several other constructs, its original correlation (0.996) was equal to the 5% quantile (0.996), and its permutation p value was 0.076, which is above 0.05. Therefore, compositional invariance was established for AI-TK as well as for the other constructs. These results indicate that compositional invariance was achieved across the high-frequency and low-frequency AI-use groups.

**Table 10:** Compositional invariance.

Constructs	Original correlation	Correlation permutation mean	5.00%	Permutation *p*-value
AIL	1	1	1	0.215
AI-TPACK	0.999	0.999	0.997	0.468
CK	1	0.999	0.998	0.828
AI-TIB	1	1	1	0.857
AI-TCK	0.999	0.999	0.998	0.085
AI-TPK	0.999	0.999	0.998	0.651
AI-TK	0.996	0.998	0.996	0.076
PCK	0.999	0.999	0.998	0.102
TSE	1	1	1	0.628
PK	0.999	0.999	0.998	0.499
PI	1	1	1	0.261

AIL, AI literacy; AI-TPACK, AI-Technological Pedagogical Content Knowledge; CK, content knowledge; AI-TIB, AI teaching innovation behavior; AI-TCK, AI-technological-content knowledge; AI-TPK, AI-technological-pedagogical knowledge; AI-TK, AI-technological knowledge; PCK, pedagogical-content knowledge; TSE, teaching self-efficacy; PK, pedagogical knowledge; PI, professional identity.

#### 4.5.2 Multi-group analysis results

This study conducted a multi-group analysis on AI-TIB to examine path coefficients across different groups after verifying the measurement invariance of the variables. The results are presented in Table 11. The bootstrap MGA results showed that, among all structural paths, only the relationship between PI and AI-TIB reached the conventional level of statistical significance between the high-frequency and low-frequency AI-use groups (*Δβ* = −0.113, *p* = 0.050). Specifically, the positive effect of PI on AI-TIB was stronger in the low-frequency group (*β* = 0.289) than in the high-frequency group (*β* = 0.176). No significant between-group differences were found for the remaining paths.

**Table 11:** Multigroup analysis.

Path	*β_(high frequency)_*	*β_(low frequency)_*	*Δβ_(high frequency –low frequency)_*	*p*	Hypotheses
AI-TK → AI-TIB	0	0.019	−0.019	0.627	Not Supported
CK → AI-TIB	0.056	0.115	−0.059	0.167	Not Supported
PK → AI-TIB	0.023	0.077	−0.054	0.247	Not Supported
PCK → AI-TIB	0.031	0.107	−0.076	0.086	Not Supported
AI-TCK → AI-TIB	0.082	0.111	−0.029	0.526	Not Supported
AI-TPK → AI-TIB	0.049	0.068	−0.019	0.660	Not Supported
AI-TPACK →AI-TIB	−0.003	0.031	−0.034	0.459	Not Supported
TSE → AI-TIB	0.317	0.223	0.094	0.149	Not Supported
PI→AI-TIB	0.176	0.289	−0.113	0.050	Supported
AIL→AI-TIB	0.327	0.292	0.035	0.549	Not Supported

β – standardized beta coefficient. AI-TK, AI-technological knowledge; CK, content knowledge; PK, pedagogical knowledge; PCK, pedagogical-content knowledge; AI-TCK, AI-technological-content knowledge; AI-TPK, AI-technological-pedagogical knowledge; AI-TPACK, AI-Technological Pedagogical Content Knowledge; TSE, teaching self-efficacy; PI, professional identity; AIL, AI literacy; AI-TIB, AI teaching innovation behavior.

## 5 Discussion

To provide a clearer overview of the empirical findings, Table 12 summarizes the results of hypothesis testing prior to the following discussion.

**Table 12:** Summary of hypothesis testing results.

Hypothesis	Statement	Result	Note
H1	Each dimension of AI-TPACK is positively associated with college teachers’ AI-TIB.	Partially supported	AI-TK → AI-TIB, AI-TPACK → AI-TIB were not significant.
H2	PI is positively associated with college teachers’ TSE.	Supported	
H3	AIL is positively associated with college teachers’ TSE.	Supported	
H4	AIL is positively associated with college teachers’ PI.	Supported	
H5	Each dimension of AI-TPACK is positively associated with college teachers’ TSE.	Partially supported	AI-TK → TSE was not significant.
H6	TSE is positively associated with college teachers’ AI-TIB.	Supported	
H7	TSE mediates the associations between AI-TPACK dimensions and AI-TIB.	Partially supported	AI-TK → TSE → AI-TIB was not supported.
H8	Each dimension of AI-TPACK is positively associated with college teachers’ PI.	Supported	
H9	PI is positively associated with college teachers’ AI-TIB.	Supported	
H10	PI mediates the associations between AI-TPACK dimensions and AI-TIB.	Supported	
H11	Each dimension of AI-TPACK is positively associated with college teachers’ AIL.	Supported	
H12	AIL is positively associated with college teachers’ AI-TIB.	Supported	
H13	AIL mediates the associations between AI-TPACK dimensions and AI-TIB.	Supported	

### 5.1 AI-TPACK and AI teaching innovation behaviors

At the level of direct effects, AI-TK and AI-TPACK did not show significant associations with college teachers’ AI-TIB, whereas the other dimensions showed significant positive associations. Thus, the corresponding hypotheses received only partial support. This finding differs from some prior studies examining teachers’ intentions to use AI. For example, Oved and Alt (2025) reported that all TPACK dimensions positively predicted teachers’ intentions to use AI. Similarly, Wang and Li (2026) found that AI-TPACK was positively associated with pre-service teachers’ behavioral intention to engage in AI-assisted teaching. However, these studies primarily focused on behavioral intention, whereas the present study adopts AI-TPACK and examines AI-TIB in authentic GenAI-supported teaching contexts, by which we mean teachers’ actual teaching work in higher education, including lesson preparation, classroom instruction, interaction with students, feedback provision, assessment design, and the practical use of GenAI tools to support these activities in real courses rather than hypothetical or decontextualized situations. Compared with usage intention, innovative behavior requires instructional redesign and sustained implementation, which place higher demands on integrative and contextualized competence. The discrepancy may therefore reflect differences in the outcome examined, as willingness to engage in AI-assisted teaching does not necessarily correspond to the generation and implementation of innovative teaching practices.

Among the direct-effect findings, the non-significant paths from AI-TK and AI-TPACK to AI-TIB are particularly noteworthy. The non-significant effect of AI-TK suggests that isolated technical knowledge does not automatically translate into innovative teaching behavior. Teachers may know how to operate GenAI tools, generate content, or use prompts at a functional level, yet such knowledge may remain instrumental unless it is connected to pedagogical purposes, disciplinary goals, and classroom decision-making. This result supports the view that technical training alone may be insufficient to promote GenAI-based teaching innovation. The non-significant direct effect of AI-TPACK is also theoretically important. A similar finding was reported by Runge et al. (2025), who found that pre-service teachers’ AI-TPACK was positively associated with perceived usefulness and perceived ease of use but showed no significant direct association with either their behavioral intention to use AI in future teaching or their actual AI use in teacher training. This evidence suggests that AI-TPACK may not be directly associated with behavioral outcomes in all contexts and that intermediate psychological processes may be relevant to how AI-related competence is reflected in teaching behavior. Although AI-TPACK represents integrative competence, this broad self-perceived knowledge structure may not directly produce innovative behavior under real instructional constraints. In practice, teachers may report that they understand how to combine AI, pedagogy, and content, but transforming this understanding into actual innovation may still depend on contextual conditions such as time, institutional support, implementation opportunities, and successful prior experience. Integrative competence may instead function more as an upstream enabling condition than as an immediate behavioral correlate (Celik, 2023).

In contrast, CK, PK, and the cross-domain dimensions involving AI and content or pedagogy showed significant positive associations with AI-TIB. These dimensions relate more directly to instructional design and decision-making and are therefore more likely to be reflected in concrete innovative practice. Overall, the present findings suggest that GenAI-based teaching innovation is associated less with isolated technical knowledge and more with the contextualized integration of AI within disciplinary and pedagogical practice. At the same time, these direct effects should not be overstated. Several statistically significant paths showed relatively small effect sizes, suggesting that even supported direct paths were modest in practical magnitude. An alternative explanation should also be acknowledged: teachers who already engage in innovative teaching may be more likely to report higher AI-related competence.

### 5.2 The relationship among professional identity, AI literacy, and teaching self-efficacy

This study found that PI is positively associated with college teachers’ TSE, supporting H2. This result aligns with Cai et al. (2022), who found that teachers’ self-efficacy is positively associated with PI. According to PIT, when teachers strongly recognize the value, significance, and social role of their profession, they develop a high sense of professional mission and responsibility. Teachers with a strong PI tend to devote more effort to exploring teaching methods (behavioral engagement) and view teaching challenges as growth opportunities through positive attribution (cognitive regulation), which is associated with enhanced TSE (Cai et al., 2022). Moreover, our study indicates that AIL is positively associated with both TSE and PI, supporting H3 and H4. This finding is consistent with recent studies (Aksit et al., 2025; Wang and Ye, 2025), which suggest that AIL is positively associated with TSE beliefs and PI development in AI-integrated educational contexts. On one hand, improved AIL allows teachers to use intelligent tools more efficiently to optimize teaching design, personalize instruction, and provide feedback, enhancing their confidence in handling teaching challenges (TSE) (Long and Magerko, 2020). On the other hand, mastering advanced technologies promotes professional growth and teaching innovation, which not only strengthens teachers’ professional competitiveness in the digital era but also deepens their understanding of the teaching role through the perceived value of leading educational change (PI) (Su, 2023). These findings suggest that promoting the combined development of technological competence and PI can stimulate teachers’ TSE and sense of professional value, supporting a positive cycle of professional growth. However, these associations should be interpreted cautiously. Because the study was cross-sectional and relied on self-report data, it is also possible that teachers with stronger professional identity or greater confidence are more willing to explore GenAI tools and therefore report higher AIL.

### 5.3 The direct associations of teaching self-efficacy, professional identity, and AI literacy with AI teaching innovation behavior

This study found that TSE, PI, and AIL were positively associated with college teachers’ AI-TIB. Thus, H6, H9, and H12 were supported. These results align with prior research (Huang et al., 2025; Lin and Chang, 2022; Wang et al., 2023). Within SCT, TSE reflects teachers’ confidence in their instructional competence. When facing the complexity and uncertainty introduced by AI, teachers with stronger TSE are more likely to view technological challenges as manageable rather than threatening. This positive efficacy belief reduces the psychological cost of experimenting with new tools and supports sustained engagement in innovative course design and classroom practice (Long and Waugh, 2018). PI is associated with innovation from an identity-based motivational perspective. Teachers who strongly identify with their professional roles are more likely to interpret AI integration as part of their professional responsibility. When technology integration aligns with professional mission, innovation becomes an identity-driven practice rather than a purely instrumental choice (Liu and Geertshuis, 2016). Finally, AIL functions as a practical competence resource. Higher AIL enables teachers to translate AI technologies into instructional strategies and classroom applications. It also reduces perceived difficulty and risk in technology use, which supports innovation in authentic GenAI-supported teaching contexts (Wang et al., 2023). Taken together, TSE, PI, and AIL can be understood as reflecting efficacy beliefs, identity-related motivation, and practical competence in relation to AI-TIB. Nevertheless, these supported paths should not be interpreted as strong standalone predictors. The effect-size results indicate that several statistically significant relationships were relatively small in practical magnitude. Thus, TSE, PI, and AIL are better understood as relatively proximal contributors within a broader multivariate system. It is also possible that teachers who already innovate subsequently report stronger AIL, PI, and TSE.

### 5.4 The mediating role of teaching self-efficacy

The results indicate that among the AI-TPACK dimensions, only AI-TK did not show a significant association with college teachers’ TSE, whereas the other dimensions showed significant positive associations. Thus, H5 received partial support. This finding differs from Rahman et al. (2022), who reported significant effects for all TPACK dimensions under the traditional framework. One possible explanation lies in the higher complexity of GenAI-supported teaching. AI-TK primarily reflects teachers’ knowledge of AI technologies, including their principles, functions, and applications (Ning et al., 2024), whereas TSE represents teachers’ overall judgment of classroom organization, instructional effectiveness, and control (Tschannen-Moran and Hoy, 2001). When technical knowledge does not align with content and pedagogy, its contribution to overall teaching confidence may remain limited. In some cases, technological complexity may even increase cognitive load and weaken perceived instructional control. In contrast, cross-domain knowledge—such as AI-TPK, AI-TCK, and integrative AI-TPACK—relates more directly to instructional design and learning optimization. These integrative capacities may strengthen teachers’ sense of control in complex teaching situations and thus show stronger associations with TSE. This finding further supports the interpretation that isolated technical knowledge is insufficient to strengthen broader instructional confidence in GenAI-supported teaching.

Further mediation analyses showed that TSE did not mediate the association between AI-TK and AI-TIB, whereas it significantly mediated the paths from the other AI-TPACK dimensions to AI-TIB. Thus, H7 received partial support. More specifically, the only unsupported TSE-mediated path was AI-TK → TSE → AI-TIB. AI-TK primarily reflects teachers’ understanding of AI technologies and their functional familiarity with AI tools (Ning et al., 2024). Such knowledge represents technical proficiency rather than instructional transformation capacity. When technical knowledge remains detached from pedagogical logic and disciplinary goals, teachers may perceive themselves as competent users of tools but not necessarily as capable designers of enhanced learning experiences. In this case, AI-TK is unlikely to activate efficacy beliefs that extend to overall instructional performance (Rahman et al., 2022). In contrast, cross-domain and integrative dimensions directly inform instructional redesign and pedagogical decision-making. These capacities reshape how teachers interpret classroom challenges and evaluate expected outcomes. As a result, efficacy beliefs operate as a translation mechanism through which integrative competence connects to innovative practice. At the same time, these indirect effects were generally small in magnitude, suggesting that TSE functions as a modest rather than powerful transmission mechanism.

### 5.5 The mediating role of professional identity

The results showed that all AI-TPACK dimensions were positively associated with college teachers’ PI, supporting H8. This finding suggests that in AI-supported contexts, integrative knowledge structures not only strengthen instructional competence but also reinforce teachers’ identification with their professional roles. Compared with traditional technological settings, AI-based teaching involves greater uncertainty and structural change. When teachers effectively integrate AI, subject content, and pedagogy, they are more likely to develop professional confidence during digital transformation and position themselves as active contributors to educational innovation. Successful experiences in managing complex instructional challenges may further strengthen their sense of professional value and mission.

Further mediation analyses showed that PI significantly mediated the associations between all AI-TPACK dimensions and AI-TIB, supporting H10. This result highlights the importance of PI in understanding how competence is connected to innovative practice. Unlike TSE, which reflects confidence in instructional effectiveness, PI represents a more stable process of identity construction. When teachers possess strong integrative competence and internalize it as part of their professional self-concept—such as viewing themselves as educators capable of leading instructional change in the GenAI era—innovative behavior becomes more sustained (Lin and Chang, 2022). AI-TPACK provides problem-solving resources, but PI determines whether these resources are activated and consistently invested in innovation. Teachers may have the necessary competence, yet without integrating AI into their sense of professional responsibility, their innovative efforts may remain occasional. When competence aligns with PI, PI functions as a stable mechanism that supports the transformation of ability into sustained innovative practice. However, this interpretation should be qualified in two ways. First, for AI-TK and AI-TPACK, the direct effects on AI-TIB were non-significant, so the corresponding PI-mediated relationships are more appropriately understood as indirect-only mediation rather than partial mediation. Second, although all PI-mediated paths were supported, their magnitudes were generally modest. It is also possible that teachers who already innovate experience stronger professional affirmation and therefore report higher PI.

### 5.6 The mediating role of AI literacy

The results showed that all AI-TPACK dimensions were positively associated with college teachers AIL, supporting H11. This finding suggests that in AI-supported contexts, integrative knowledge structures provide a foundation for developing systematic understanding and application of AI (Ning et al., 2024). Unlike isolated technical knowledge, AI-TPACK emphasizes the coordinated integration of AI technology, subject content, and pedagogy. Such structural knowledge not only enhances teachers’ understanding of AI functions but also strengthens their ability to make appropriate and optimized instructional decisions in specific teaching situations. When teachers interpret the educational value of AI within a multidimensional knowledge framework, AIL is more likely to develop in a stable and sustained manner.

Further mediation analyses showed that AIL significantly mediated the associations between all AI-TPACK dimensions and AI-TIB, supporting H13. This result points to the role of practical AI-related competence in understanding innovative practice. Unlike TSE, which emphasizes efficacy beliefs, and PI, which reflects identity construction, AIL represents teachers’ comprehensive ability to understand, evaluate, and apply AI technologies in authentic GenAI-supported teaching contexts (Niswah and Dewi, 2024). When integrative knowledge structures translate into operational and contextual application skills, teachers are better equipped to implement innovative practices in course design, classroom interaction, and assessment. AI-TPACK provides a cognitive framework, whereas AIL reflects its practical enactment. If teachers possess theoretical integration knowledge but lack contextual application experience, innovative behavior may remain unstable. In contrast, as teachers accumulate experience in lesson preparation, instruction, and evaluation using GenAI tools, AIL strengthens. This development reduces perceived uncertainty and operational risk, thereby supporting more sustained innovative practice (Wang et al., 2023). Compared with TSE and PI, AIL appeared to provide the relatively stronger indirect pathway in the model. Even so, these indirect effects remained modest rather than large, and for AI-TK and AI-TPACK the supported AIL-mediated relationships are more appropriately interpreted as indirect-only mediation because the corresponding direct effects on AI-TIB were non-significant.

### 5.7 Multi-group analysis of AI teaching innovation behavior

The multi-group analysis should be interpreted cautiously. The multi-group analysis revealed a borderline significant difference between the high-frequency and low-frequency groups in the path from PI to AI-TIB. This association was stronger in the low-frequency group and weaker in the high-frequency group, and no other path differences reached statistical significance. This pattern does not support broad claims about identity reconstruction. From the perspective of SCT, behavior depends not only on internal beliefs and identity but also on experiential feedback (Bandura, 1982). One possible explanation is that for teachers in the low-frequency group, PI may play a compensatory motivational role when routine GenAI use is still limited. In contrast, for teachers in the high-frequency group, innovative behavior may depend less on PI alone because repeated GenAI use already provides more direct experiential support for action. However, because this group difference was only borderline significant and all remaining path differences were non-significant, this interpretation should be treated as tentative. At most, the MGA findings provide preliminary evidence of a narrow group difference that should be verified in future research.

## 6 Implications

### 6.1 Theoretical implications

First, this study applies the AI-TPACK framework to AI-supported teaching and examines its association with AI-TIB. Unlike traditional TPACK research that emphasizes technology use or integration competence, this study focuses on innovative practice in authentic teaching contexts. The findings show that AI-TPACK dimensions differ in their associations with innovation, suggesting that AI-TPACK may be better understood as a set of differentiated sub-competencies rather than a single aggregate resource when explaining AI teaching innovation behavior.

Second, this study tests the mediating roles of TSE, PI, and AIL between AI-TPACK and AI-TIB. The results suggest that the association between competence structure and innovation is linked to multiple psychological and capability-based factors rather than a single pathway. In particular, the relatively stronger role of AIL suggests that practical AI-related competence may be more proximal to AI-TIB than upstream knowledge dimensions alone. These findings enrich the understanding of how technology integration competence translates into innovative practice and demonstrate the usefulness of SCT and PIT as interpretive frameworks in GenAI-supported education, rather than claiming a substantive extension of these theories.

Finally, the multi-group analysis yielded only one borderline group difference, and no other path differences were significant. The MGA findings should therefore be interpreted cautiously. Rather than indicating a broad process of identity reconstruction, they provide only tentative evidence that the role of PI in innovation may vary somewhat with teachers’ level of practical engagement with AI.

### 6.2 Practical implications

Based on the findings, the present study offers several evidence-informed suggestions for supporting college teachers’ AI-TIB. First, institutions may prioritize the development of integrative competence in AI contexts. Because AI-TPACK dimensions differ in their associations with innovation, training should move beyond technical operation and emphasize contextualized integration. Universities may organize AI-focused instructional design workshops that guide teachers through the full process of goal setting, tool selection, activity design, and assessment optimization within authentic teaching tasks. Problem-oriented redesign can help teachers develop systematic integrative capacity rather than isolated technical skills. This suggestion is especially relevant because the findings showed that isolated technical knowledge alone was insufficient to predict AI-TIB directly.

Second, institutions may place particular emphasis on strengthening teachers’ AI literacy, because AIL showed the comparatively strongest association with AI-TIB in the model, although the corresponding effects were still modest rather than large. Accordingly, professional development may focus not only on tool use, but also on ethical evaluation, contextual application, and pedagogical adaptation of GenAI in teaching. Universities may also showcase successful AI teaching cases and establish peer-sharing platforms to strengthen teachers’ confidence in their instructional capability. Clarifying teachers’ professional roles in digital transformation may further reinforce the perception that AI integration forms part of professional responsibility, thereby helping to sustain innovation motivation.

Third, institutions may create opportunities for sustained practice to support the development of AIL. Providing low-risk experimental environments, such as pilot AI classrooms or small-scale innovation projects, can help teachers accumulate experience in authentic contexts. Continuous practice and feedback may help teachers to convert integrative knowledge into stable innovative performance. These recommendations should nevertheless be interpreted cautiously in light of the associative nature of the findings.

Finally, the multi-group results provide only provisional support for differentiated support. Teachers with limited AI experience may require foundational operational guidance and feedback support, whereas experienced teachers may benefit from advanced innovation resources, such as interdisciplinary collaboration projects or teaching innovation grants. Layered support strategies may help support AI-TIB across different developmental stages. Because this recommendation rests on only one borderline group difference, it should be interpreted cautiously and treated as tentative until further evidence becomes available.

## 7 Limitations and future research directions

Although this study examined the relationships between AI-TPACK dimensions and college teachers’ AI-TIB and assessed multiple indirect pathways, several limitations warrant further investigation.

First, this study used a cross-sectional design, which limits conclusions about temporal dynamics among competence structures, psychological factors, and innovative behavior. In rapidly evolving AI environments, AIL and PI may change over time. Moreover, the directional relationships among AIL, PI, and TSE should be interpreted with caution, as reciprocal associations may also be possible. Longitudinal or staged intervention studies could provide deeper insight into how competence is related to innovation across developmental phases.

Second, the study employed a convenience sampling strategy, and the sample was characterized by a relatively high proportion of female and younger teachers. In addition, participants were drawn from a limited number of higher education institutions. These characteristics may reduce the representativeness of the sample and limit the generalizability of the findings. Because participation was voluntary, self-selection bias may also have occurred, such that teachers who were more interested in AI, more confident in their digital competence, or more positively disposed toward innovation may have been more likely to respond. Teachers’ AI-related competencies and innovation behaviors may also vary across institutional types, regional contexts, and disciplinary backgrounds. The findings should therefore be interpreted cautiously and should not be generalized directly to all higher education contexts. In particular, the study was conducted only in China, and the single-country context may limit the transferability of the findings to higher education systems with different institutional structures, professional norms, or levels of AI adoption. In addition, the exclusion of responses with more than 80% extreme-category answers may have removed some genuinely strong opinions, even though this criterion was intended to reduce potential floor or ceiling effects and improve response quality. Future research could examine the robustness of the findings by comparing results obtained with and without this screening rule, or by applying alternative response-quality thresholds. Future research should employ more diverse and representative sampling strategies, include cross-national comparisons where possible, and examine potential variations across different university types, regions, and academic disciplines.

Third, the study relied primarily on self-report measures, which may involve social desirability or common method bias. Although common method bias was examined in the main Results section and the statistical tests did not indicate severe bias, the use of a single self-report survey remains a methodological constraint, especially because the focal constructs were collected from the same respondents at the same time. Future studies may incorporate multiple data sources, such as classroom observations, instructional product evaluations, peer assessments, or student feedback, to enhance objectivity and robustness. In addition, although the AI-TIB scale was administered with a scenario-based instruction that anchored responses to teaching in a GenAI environment, the original item wording was retained from a general innovation scale. Therefore, some responses may still have reflected general innovative behavior rather than AI-specific teaching innovation. Future research may strengthen construct specificity by developing or validating item wording that more explicitly refers to AI-supported instructional innovation.

Fourth, the study did not include institutional-level controls, such as differences in university policy support, digital infrastructure, AI training provision, or organizational climate for teaching innovation. These factors may shape teachers’ opportunities to develop AI-related competence and translate it into practice. In addition, because respondents were nested within cooperating universities, the assumption of full independence among observations may not have been completely satisfied. Future research may benefit from collecting more detailed institutional data and applying multilevel modeling to distinguish teacher-level and institution-level effects.

Fifth, in the multi-group analysis, AI usage frequency was categorized into “high” and “low” groups. This binary classification may be relatively crude, as it combines participants with varying usage frequencies (e.g., “several times a month” and “never”) into the same group, potentially weakening group differences and losing nuanced information. Future research may adopt more refined grouping strategies, such as multiple categorical divisions or latent class analysis, to improve classification accuracy and better capture the effects of AI usage intensity on teachers’ AI teaching innovation behavior. Moreover, future studies may examine whether discipline moderates the proposed model, as teachers in humanities, engineering, medical, arts, and physical education contexts may differ in their opportunities, constraints, and pedagogical uses of GenAI.

## 8 Conclusion

This study integrated AI-TPACK framework, SCT, and PIT in GenAI-supported teaching contexts and proposed a multiple mediation model. Using self-reported survey data from 898 college teachers recruited through a convenience sample, we examined the associations between AI-TPACK dimensions and AI-TIB, as well as the roles of TSE, PI, and AIL. The results showed that AI-TK and integrative AI-TPACK did not have significant direct associations with AI-TIB, whereas the other AI-TPACK dimensions did. All AI-TPACK dimensions were positively associated with PI and AIL, and all except AI-TK were positively associated with TSE. PI and AIL showed both direct associations with AI-TIB and significant mediating roles, whereas the mediating role of TSE received partial support. Furthermore, multi-group analysis indicated that PI showed a borderline stronger association with AI-TIB in the low-frequency AI-use group, although this finding should be interpreted cautiously. Overall, the findings point to a theoretically specified pattern of direct and indirect associations among AI-TPACK dimensions, TSE, PI, AIL, and AI-TIB. Given the sample and design, these findings should be interpreted as evidence from self-reported data provided by college teachers in a convenience sample. Most importantly, GenAI-supported teaching innovation appears to depend less on isolated AI technical knowledge and more on pedagogically and disciplinarily embedded AI competence, AI literacy, professional identity, and efficacy beliefs.

## Data Availability

The raw data supporting the conclusions of this article will be made available by the authors, without undue reservation.
