# Supplementary material for: AI teaching innovation behavior among college teachers: a structural equation modeling analysis based on the AI-TPACK framework, teaching self-efficacy, professional identity, and AI literacy
Source: Front Psychol. 2026 Sep 7;17:1834827. doi: 10.3389/fpsyg.2026.1834827 (PMC13590781; doi:10.3389/fpsyg.2026.1834827)
Supplement: Supplementary file 1 [file Table_1.DOCX]

Table S1. Effect Sizes (f²) for Structural Paths in the PLS-SEM Model

|  | AIL | AI-TPACK | CK | AI-TIB | AI-TCK | AI-TPK | AI-TK | PCK | TSE | PK | PI |
| --- | --- | --- | --- | --- | --- | --- | --- | --- | --- | --- | --- |
| AIL |  |  |  | 0.145 |  |  |  |  | 0.101 |  | 0.160 |
| AI-TPACK | 0.106 |  |  | 0.000 |  |  |  |  | 0.005 |  | 0.038 |
| CK | 0.036 |  |  | 0.018 |  |  |  |  | 0.008 |  | 0.006 |
| AI-TIB |  |  |  |  |  |  |  |  |  |  |  |
| AI-TCK | 0.042 |  |  | 0.020 |  |  |  |  | 0.021 |  | 0.015 |
| AI-TPK | 0.046 |  |  | 0.009 |  |  |  |  | 0.015 |  | 0.023 |
| AI-TK | 0.061 |  |  | 0.000 |  |  |  |  | 0.001 |  | 0.012 |
| PCK | 0.035 |  |  | 0.012 |  |  |  |  | 0.005 |  | 0.072 |
| TSE |  |  |  | 0.096 |  |  |  |  |  |  |  |
| PK | 0.094 |  |  | 0.005 |  |  |  |  | 0.02 |  | 0.029 |
| PI |  |  |  | 0.086 |  |  |  |  | 0.106 |  |  |

*Note:* *AIL: AI literacy, AI-TPACK: AI-Technological Pedagogical Content Knowledge, CK: Content knowledge, AI-TIB: AI teaching innovation behavior, AI-TCK: AI-technological-content knowledge, AI-TPK: AI-technological-pedagogical knowledge, AI-TK: AI-technological knowledge, PCK: pedagogical-content knowledge, TSE: teaching self-efficacy, PK: pedagogical knowledge, PI: professional identity.*
